# Fear Conditioning to Subliminal Fear Relevant and Non Fear Relevant Stimuli

**DOI:** 10.1371/journal.pone.0099332

**Published:** 2014-09-08

**Authors:** Ottmar V. Lipp, Clare Kempnich, Sang Hoon Jee, Derek H. Arnold

**Affiliations:** 1 School of Psychology and Speech Pathology, Curtin University, Perth, Western Australia, Australia; 2 Science of Learning Research Centre, The University of Queensland, St. Lucia, Queensland, Australia; 3 School of Psychology, The University of Queensland, St. Lucia, Queensland, Australia; Tokai University, Japan

## Abstract

A growing body of evidence suggests that conscious visual awareness is not a prerequisite for human fear learning. For instance, humans can learn to be fearful of subliminal fear relevant images – images depicting stimuli thought to have been fear relevant in our evolutionary context, such as snakes, spiders, and angry human faces. Such stimuli could have a privileged status in relation to manipulations used to suppress usually salient images from awareness, possibly due to the existence of a designated sub-cortical ‘fear module’. Here we assess this proposition, and find it wanting. We use binocular masking to suppress awareness of images of snakes and wallabies (particularly cute, non-threatening marsupials). We find that subliminal presentations of both classes of image can induce differential fear conditioning. These data show that learning, as indexed by fear conditioning, is neither contingent on conscious visual awareness nor on subliminal conditional stimuli being fear relevant.

## Introduction

It has been suggested that human evolution has resulted in a sub-cortical ‘fear module’, which is preferentially activated by stimuli that were fear relevant in our evolutionary context, such as snakes, spiders and angry human faces [1]. Moreover, possibly because this module is thought to be sub-cortical and centered on the amygdala, this activation seems to be relatively impervious to manipulations that suppress conscious visual awareness. This proposal is consistent with the observation that pictures of feared animals will elicit the same level of electrodermal responding, an index of sympathetic activation, in phobic participants regardless of whether they are presented clearly visible or backwardly masked [2]. Using binocular masking, fMRI studies suggest that the amygdala is reactive to fearful faces that are suppressed from awareness, but unresponsive to suppressed happy faces [3]. Similarly, fearful expressions reportedly escape the effects of binocular masking *sooner* (i.e., at lower levels of signal strength) than do faces expressing other emotions [4], [5]. These findings have suggested a utility for masking procedures to address the vexed question of whether consciousness is a pre-requisite for human learning, as evidenced by fear conditioning - for a recent review see [6].

Earlier studies have used backward masking as a means to achieve this. In a typical differential human fear conditioning experiment, one conditional stimulus (CS+) is paired with an electrocutaneous shock (the unconditional stimulus), which is set by the participant to an intensity that is “unpleasant, but not painful”, whereas a second conditional stimulus is presented alone (CS−). Learning is indexed by larger electrodermal responses in response to CS+ than to CS− during periods that may, or may not, precede an unconditional stimulus presentation. Backward masking of the conditional stimuli was applied either during conditioning [7], [8] or during subsequent extinction training [9], [10]. On masked trials, conditional stimuli were presented for 30 ms and followed by pattern masks, presented for 100 ms, whereas the conditional stimuli were presented for 130 ms on unmasked trials. During acquisition, the shock unconditional stimulus was presented 500 ms after conditional stimulus onset. Consistent with the predictions of the fear module account [1], differential fear conditioning as indexed by electrodermal responses was evident to fear relevant stimuli, snakes, spiders or angry faces, when presented masked, but not to fear irrelevant stimuli, such as flowers, mushrooms or happy faces.

Given the slow latency of electrodermal responses, which is in excess of 1 second, conditioning paradigms that utilize backward masking and short CS-US intervals are not optimal. They differ considerably from the more customary longer delay conditioning procedures in which the conditional stimuli are presented for six or eight seconds and followed by the unconditional stimulus. The longer delay interval permits the observation of physiological responses before the unconditional stimulus is presented. Paradigms in which the unconditional stimulus follows the conditional stimulus within 500 ms necessitate the inclusion of unpaired (CS+ alone) test trials during acquisition to avoid confounding conditional and unconditional responses or the assessment of masked conditioning during extinction training which may lead to generalization decrements due to stimulus change.

Binocular masking has become a popular tool when investigating responses to subliminal stimuli, as it can be used to *persistently* suppress awareness for periods of seconds. In binocular masking, awareness of usually salient images presented selectively to one eye is suppressed by presenting a high-contrast mask to the other eye. It is achieved by either intermittently flashing different masking images to one eye [11] or by intermittently switching a to-be-suppressed image and a mask between the eyes [12]. A recent study [13] used binocular masking to suppress awareness of fear relevant conditional stimuli, a male and a female fearful face. They found differential electrodermal responding regardless of whether participants had been consciously aware of the fearful faces. These results suggest people need not be aware of the content of a conditional stimulus for conditioning to occur. Moreover, these data are at least *consistent* with a special role for fear relevant stimuli, as the successful subliminal conditioning was elicited in response to a subliminal fearful face. However, these data are ambiguous in this last regard, as the study did not encompass a condition in which fear was conditioned to a non-fear relevant conditional stimulus. Thus, it is possible that a non-threatening subliminal conditional stimulus might have been equally effective.

**Figure 1:**
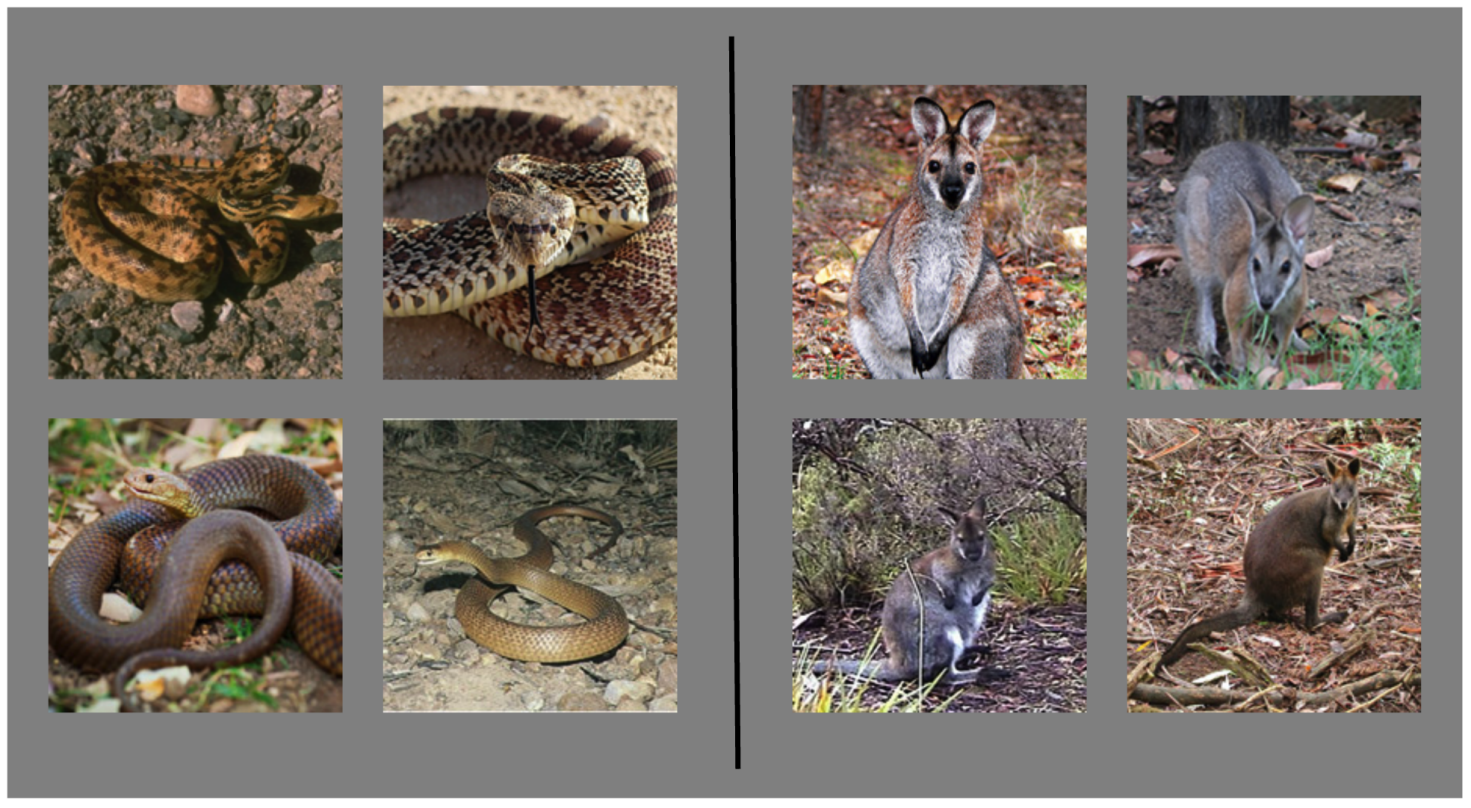
Depiction of images used as conditional stimuli.

In the current study we use pictures of animals to assess whether subliminal fear conditioning is limited to fear-relevant stimuli. To minimize picture specific discrimination effects, four different pictures of snakes and four different pictures of wallabies (small, cuddly, completely non-threatening marsupials, see Figure 1) were presented as conditional stimuli in a differential fear conditioning procedure. As fear-relevant stimuli we used pictures of fear-relevant animals, rather than emotional faces, as supraliminal fear conditioning to the latter has been shown to be subject to verbal instruction [14], whereas fear conditioning to the former is not - for a review see [15]. Conditioning was assessed across four blocks of trials. Blocks one and three presenting the conditional stimulus images in the clear whereas they were masked using binocular switch suppression [12] in blocks two and four. If subliminal fear conditioning is limited to fear-relevant animals, we should observe differential conditioning to subliminal snake images, but not to subliminal pictures of harmless wallabies. To preface our results, we find that both groups of images do not differ in their efficiency to support subliminal fear conditioning.

## Materials and Methods

Ethical approval for the experiment was obtained from the University of Queensland Ethics Committee. All participants were informed that they could withdraw from the Experiment at any time without penalty, and provided written consent to participate in the experiment after they had read an information sheet describing the experimental procedure. Thirty-two participants (21 female; mean age: 20.52 years, SD = 2.44) participated for course credit and were assigned to one of two groups (CS+: Snake or Wallaby). The two groups did not differ in age, t(28) = 0.728, ns. Electrodermal data from two participants were unusable, as they did not show electrodermal responses.

**Figure 2:**
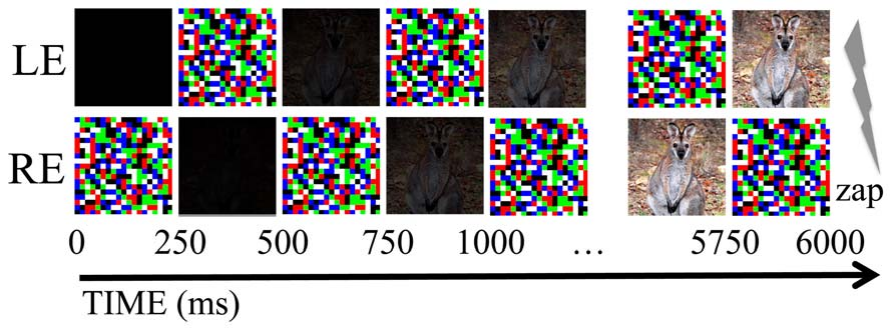
Depiction of the masked image presentation protocol. During presentations a to-be-masked image of a snake or wallaby alternated with a colored mask between being presented to either eye at a rate of 2 Hz. The brightness of the to-be-masked image was ramped on, from black to full brightness, across the first 3 seconds of the presentation, meaning that CS were presented at full intensity for the final 3 seconds of each 6 second test presentation, culminating in a shock for CS+ presentations.

Conditioning training consisted of four blocks of eight trials. In each block images of four snakes and four wallabies (see Figure 1) were presented in random order. In masked trials a colored noise Masking image and a Test image alternated between being presented to either eye for periods of 250 ms – a method known as binocular switch suppression [12], see Figure 2. Alternating presentations persisted for 6 seconds. There was then a 1–3 second inter-stimulus-interval before the next trial commenced. The luminance of Test images was linearly ramped (from black to full brightness) over the first three seconds of each stimulus presentation, meaning that Test images were presented at full contrast for the final 3 seconds/12 alternations of each presentation. Subjectively, throughout the entire experiment, when masking was sufficient the participant only reported seeing the masks, and was unaware of the Test image presentations. Unmasked presentations differed only in that Test images were presented to both eyes (and unmasked) for 6 seconds.

During blocks one and three, Test images were unmasked, whereas in blocks two and four pictures were masked. For half of the 30 participants, the Snake Group, a shock US was presented at the conclusion of each 6-second snake image presentation (CS+) whereas pictures of wallabies were presented alone (CS−). For participants in the Wallaby Group the shock US was presented after presentations of wallaby images (CS+) whereas the snake images (CS−) were presented alone. The shock US had been set by each participant individually to an intensity that was reported to be ‘unpleasant, but not painful’ prior to conditioning training, and it was presented after the CS+ during unmasked and masked blocks. After completion of the conditioning sequence, participants were questioned as to whether they had seen the animal pictures during masked trial blocks. To formally assess sensitivity, the experiment concluded with a manipulation check, a signal detection experiment, in which participants were presented with 40 masked trials. These consisted of five presentations of each of the eight animal pictures. On each trial during this procedure participants were asked to indicate whether the masked image presentation had been of a snake (the signal). This was done at the end of the experiment in order to maximize sensitivity to any learning that might have occurred during the preceding conditioning blocks of trials. We did not ask participants to make trial-by-trial reports as to image content during conditioning blocks, as we did not want to confound our measure of implicit conditioning with anticipatory ‘guesses’.

Electrodermal responses were recorded with a Biopac MP150 recording unit and scored as the largest response that started within 4 to 7 s after CS onset. Responses in this latency window capture the anticipation of the unconditional stimulus without contamination from the unconditional response that this stimulus will elicit [16]. Responses were averaged across the four trials per CS+ and CS− presented in each block and subjected to a 2×2×2×2 (Group [Snake vs. Wallaby] × CS [CS+ vs. CS−] × Presentation condition [Masked vs. Not masked] x Block [1 vs. 2]) factorial mixed model ANOVA with repeated measures on the last three factors. Level of significance was set to .05 and partial eta squared, η_p_^^2^^, is reported as a measure of effect size.

## Results

**Figure 3:**
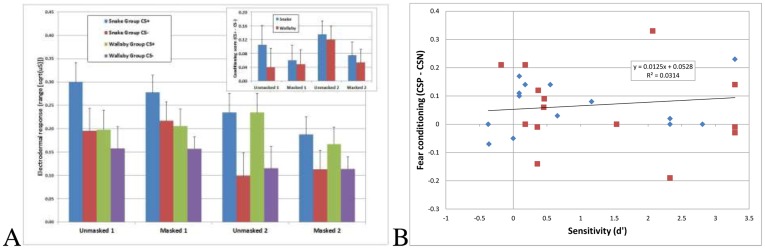
Electrodermal responses to masked and unmasked stimulus presentations (A) and scatter plot of d′ scores in a classification task (snake/wallaby) for masked images against individual conditioning scores for masked trials (B). CS+ electrodermal responses (A) are heightened relative to CS- responses. This is true regardless of whether the CS+ was a snake (blue and red bars) or a wallaby (green and purple bars). This was also true across all four blocks of trials, including blocks of masked image presentations. The insert depicts conditioning scores (response to CS+ - response to CS-) as a function of Group and Block. Error bars depict +1 SEM. The scatterplot of individual conditioning scores against classification sensitivity (B) reveals no correlation, showing that people did not have to be aware of the species depicted in the masked image in order to display differential fear conditioning.

Figure 3A shows electrodermal responses elicited by CS+ and CS− in anticipation of the unconditional stimulus during unmasked and masked blocks of trials for the Snake and Wallaby Groups. Electrodermal responses were larger to CS+ than to CS− in both groups, regardless of whether the CSs+ were of snakes or wallabies (F(1,28) = 21.19, p<.001, η_p_^^2^^ = .431) and declined across successive experimental blocks (F(1,28) = 9.14, p = .005, η_p_^^2^^ = .246). The analysis also revealed a marginal Group x Block interaction (F(1,28) = 3.34, p = .078, η_p_^^2^^ = .106) reflecting that the decline across blocks was significant in Group Snake (F(1,28) = 11.76, p = .002, η_p_^^2^^ = .296) but not in Group Wallaby (F(1,28) = 0.72, p = .405, η_p_^^2^^ = .025). All other terms were not significant (all F(1,28)<1.60, p>.210, η_p_^^2^^<.054). Most important in this context is that differential fear conditioning did not differ between unmasked and masked blocks of trials and did not differ between groups. A similar pattern of results emerged if we limited the analysis to participants in Group Wallaby. The main effect for conditioning was significant (F(1,14) = 5.58, p = .033, η_p_^^2^^ = .285), but not other term reaching the level of significance (all F(1,14)<2.43, p>.140, η_p_^^2^^<.150).

Of our 30 participants, 12 had reported post-hoc having seen ‘*something*’ other than a mask on at least one of the masked trials, whereas 18 reported having had no such impression. An analysis of electrodermal data from masked blocks of trials for the 18 participants who had reported *not* having seen anything (nine per group) still yielded a main effect for conditioning (F(1,16) = 5.79, p = .029, η_p_^^2^^ = .266) which was not qualified by an interaction, all F(1,16)<1.0, p>.37, η_p_^^2^^<.050.

Data from the post conditioning manipulation check, a signal detection experiment in which participants were presented with 40 masked trials and had to indicate whether the animal displayed was a snake, were scored as hits and false alarms, and converted into d′ scores. The d′ measure combines hit rates and false alarm rates to provide a measure of sensitivity to the stimuli that are presented (for more details see 17). These ranged from −.37 to 2.33 (Mean = .925; SD = 1.0 – data for one participant were lost due to experimenter error). This indicates that some participants were able to identify the content of masked images reliably, consistent with subjective reports. This is not unexpected when using binocular masking, as people are differentially susceptible to binocular masking and failures of masking can result from a failure to maintain focus and from eye blinks. Participants who reported post-hoc seeing ‘*something*’ during conditioning obtained higher d′ scores (M = 1.95, SD = 0.79) than those who reported seeing nothing (M = .30, SD = 0.47; t(27) = 7.04, p<.001), however, d′ in the latter group was significantly different from zero (t(17) = 2.69, p = .015).

These analyses confirm, however, that a large number of our participants evidenced no subjective sensitivity to the content of masked images, as indexed by guessing if the masked image had been of a snake or a wallaby. Sixteen of the 29 participants who provided valid data had a d′ of .5 or lower. Moreover, in Figure 3B we have plotted sensitivity scores against differential fear responding (electrodermal response to CS+ - electrodermal response to CS−) on masked blocks of trials in participants from the Snake (red squares) and Wallaby (blue diamonds) Groups. There was no difference in d′ between these groups (t(27) = 0.569, ns) and the extent of fear conditioning was independent of subjective sensitivity as measured in our manipulation check.

## Discussion

The current results replicate previous reports of fear conditioning to stimuli hidden from conscious awareness via backward masking [7]–[10] or by simultaneous flash suppression [13]. However, overall we find no difference in the extent of conditioning when participants were trained with snake CSs+ or with wallaby CSs+. Although Figure 1 may suggest otherwise, this also held for the differential responses observed in the first unmasked block of training. The apparent difference in differential responding between the two groups is likely to reflect on selective sensitization [18] – enhanced autonomic responding to high salience stimuli (pictures of snakes) relative to low salience stimuli (pictures of wallabies) after shock workup. This non-associative effect enhances the response to the CS+ in Group Snake and the response to the CS− in group Wallaby, enhancing or reducing the respective differential conditioning effects. This difference dissipates as sensitization wears off at later stages of the experiment. Overall, the current result is inconsistent with the notion that fear conditioning to images of fear-relevant inputs has a special status with regards to the suppression of visual awareness, as a CSs+ consisting of a group of harmless, rather cute, marsupials was equally efficacious. These findings are not consistent with the predictions made by the fear module account [1] and add to the increasing number of reports that question the preferential processing of fear-relevant stimuli, be they pictures of animals or of emotional faces.

One might argue that this finding reflects on the observation that emotional stimuli, be they emotional a-priori or as a result of conditioning, are likely to dominate in binocular rivalry displays [19], [20]. This is not supported by our behavioural data as pictures of CS+ were not recognized correctly more often than pictures of CS−. However, future research needs to provide a more fine grained analysis to exclude this option. A second limitation of the present report is that although we are able to state that there was no difference in conditional responding to masked and unmasked pictures of snakes and wallabies, we cannot state that the conditioning observed in the two groups was similar. A Bayesian analysis of the skin conductance data in response to CS+ Snakes and CS+ Wallabies, restricted to the 18 participants who evidenced no subjective sensitivity to these stimuli during masking, revealed 95% CIs for the difference between the group means that extended from −0.134 to +0.223. As zero is within this range, we cannot reject the null hypothesis that these CS+ stimuli are equally effective. The effect size, however, has 95% CIs extending from −0.71 to +1.25, so we cannot assert the similarity of these stimuli with any greater confidence than offered by our failure to reject the null hypothesis following standard statistical analyses.

Readers should note that for some of our participant binocular masking was ineffective. However, for others it was effective to the extent that they evidenced no sensitivity when making subjective categorizations after having completed four blocks of conditional trials, throughout which they had displayed differential electrodermal responding. Moreover, there was no correlation between the extent of fear conditioning and the degree of subjective sensitivity to masked stimuli in a final manipulation check. In sum, we have strong evidence for fear conditioning to subliminal inputs, but this was not contingent on those inputs being fear relevant.

While our data show that participants do not have to be aware of the content of masked images for fear conditioning to occur, we would urge caution when interpreting these data. Sensitivity to suppressed input was indexed by asking participants if the masked input had been an image of a snake or a wallaby, but it is entirely possible that the differential fear conditioning that took place was not contingent on this discrimination. Clearly the visual system was able to distinguish these two classes of input on some basis at some level of processing, but this need not imply that the discriminant basis during masked trials involved a subliminal recognition of a wallaby or a snake. Rather, any distinguishing characteristic, however subtle, might have served. Simply put, while subjective sensitivity was indexed by having participants attempt to categorize masked inputs as snakes or wallabies, the neural locus responsible for differential conditioning might not have been distinguishing inputs on this basis, potentially explaining the dissociation between our measures of subjective sensitivity and subliminal conditioning. We believe these comments hold for other demonstrations of subliminal learning [7]–[10], [13].

Having acknowledged that our evidence for subliminal fear conditioning might not have involved a neural locus capable of object recognition, we would like to stress that we nonetheless have demonstrated a segregation between differential fear conditioning and awareness of whether the conditional stimulus on a particular trial was a CS+ or a CS−. As unmasked trial blocks always preceded masked blocks, participants were perfectly aware which conditional stimulus class was the CS+, but many participants displayed no subjective sensitivity when attempting to identify the masked input class despite having displayed differential electrodermal responding for these classes of input. Hence we are justified in describing this as evidence for subliminal fear conditioning, and as evidence of learning in the absence of awareness. We would not like to suggest, however, that participants could not possibly learn to distinguish these masked inputs. It seems probable that with sufficient training with feedback, participants might learn to recognize the possibly subtle stimulus characteristics upon which differential conditioning was based. At minimum, it seems reasonable to suggest that they might learn to recognize their own heightened autonomic responding to masked CS+ inputs. Assessing these caveats, however, lies beyond the scope of this paper, and they do not undermine our central observation, that in our experiment participants displayed differential fear conditioning to stimuli that they could not reliably distinguish in a subjective task.

The main point we wish to make is that while fear conditioning can be induced by stimuli to which participants display no subjective sensitivity, this is not absolutely contingent on the subliminal stimuli being fear relevant. Non-threatening images of cute marsupials have proven equally effective for this purpose.
